# Age-Specific Differences in the Dynamics of Neutralizing Antibody to Emerging SARS-CoV-2 Variants Following Breakthrough Infections: A Longitudinal Cohort Study

**DOI:** 10.3390/vaccines13101013

**Published:** 2025-09-28

**Authors:** Zhihao Zhang, Xiaoyu Kang, Xin Zhao, Sijia Zhu, Shuo Feng, Yin Du, Zhen Wang, Yingying Zhao, Xuemei Song, Xinlian Li, Hao Cai, Meige Liu, Pinpin Long, Yu Yuan, Shanshan Cheng, Chaolong Wang, Guoliang Yang, Sheng Wei, Tangchun Wu, Jianhua Liu, Li Liu, Hao Wang

**Affiliations:** 1 Ministry of Education and State Key Laboratory of Environmental Health (Incubating), School of Public Health, Huazhong University of Science and Technology, Wuhan 430030, China; m202275468@hust.edu.cn (Z.Z.); kangxiaoyu2002@163.com (X.K.); wut@mails.tjmu.edu.cn (T.W.); 2 Yichang Center for Disease Control and Prevention, Yichang 443000, China; lxcuut0204@163.com (X.Z.);; 3 Oxford Vaccine Group, Department of Paediatrics, University of Oxford, Oxford OX1 2JD, UK; 4 The Central Hospital of Wuhan, Tongji Medical College, Huazhong University of Science and Technology, Wuhan 430030, China; 5 School of Public Health and Emergency Management, Southern University of Science and Technology, Shenzhen 518055, China

**Keywords:** SARS-CoV-2, breakthrough infection, dynamics of NAb, age-specific

## Abstract

**Background**: The continuous evolution of SARS-CoV-2 necessitates the development of targeted strategies based on the immunological profiles of distinct age groups. Despite this imperative, comprehensive insights into the dynamics and broad-spectrum efficacy of neutralizing antibodies (NAbs) against emerging variants across different age groups, particularly in children, remain inadequate. **Methods**: Following the termination of China’s dynamic ‘zero-COVID-19’ policy in January 2023, which coincided with a widespread Omicron outbreak and numerous breakthrough infections, a longitudinal cohort study was established encompassing all age groups in Hubei, China. Follow-up assessments were conducted in March (Visit 1), June (Visit 2), and October (Visit 3) 2023. A total of 320 individuals were randomly selected and stratified into three age categories: children (<18 years, n = 80), adults (18–59 years, n = 167), and the elderly (≥60 years, n = 73). The NAbs against emerging SARS-CoV-2 variants BA.5, XBB.1.5, EG.5, and JN.1 were evaluated for each group. Trajectory modeling was employed to classify antibody trends into five categories: low-level stability, median-level stability, high-level stability, early increase, and late increase. **Results**: In March 2023, children exhibited significantly higher NAb levels against BA.5, XBB.1.5, EG.5, and JN.1 compared to adults and the elderly. However, these levels rapidly declined. From June to October 2023, no significant difference in NAb levels was observed between children and the other age groups. Regarding the broad-spectrum effectiveness of NAbs, the effectiveness in children was comparable to that of adults and the elderly in March 2023. However, from June to October 2023, children’s effectiveness became significantly lower than that of the other age groups. Trajectory analysis revealed that the highest proportions of high-level stability (31.3%) and median-level stability (42.5%) were observed among children. In contrast, adults and the elderly were most commonly categorized into the early increase (adult 46.7%, elderly 49.3%) and median-level stability (adult 22.1%, elderly 20.5%) categories. **Conclusions**: Although children initially demonstrate higher levels of NAbs, these levels decrease more rapidly than in adults and the elderly, eventually equalizing in later stages of recovery. Furthermore, the broad-spectrum effectiveness of NAbs in children is narrower than in other age groups. These findings suggest that children are at an elevated risk of infection with newly emerging variants, underscoring the urgent need to intensify focus on reinfections among children and develop tailored strategies to protect this vulnerable population.

## 1. Introduction

The SARS-CoV-2 pandemic has imposed a substantial global disease burden, with over 776 million reported cases and approximately 7.06 million deaths as of September 2024 [1]. Despite the World Health Organization no longer designating COVID-19 as a global public health emergency, the continuous evolution of SARS-CoV-2 leading to immune escape has driven waves of reinfection [2]. These persistent challenges pose a substantial threat to global public health and raise concerns regarding the risk of future infection [3].

The risk of SARS-CoV-2 infection and the severity of post-infection outcomes vary by age group amid emerging variants [4,5]. Children have milder post-infection symptoms, while elderly individuals experience prolonged viral positivity and more severe consequences upon reinfection [6,7,8,9,10,11]. A critical factor driving these age-related differences is the variability in neutralizing antibody (NAb) levels across age groups. The neutralization of emerging variants significantly influences both SARS-CoV-2 infection likelihood and COVID-19 severity [12,13]. Previous studies have explored antibody responses following SARS-CoV-2 infection across different age groups, often finding lower antibody levels in older adults [14,15,16]. However, these investigations have primarily concentrated on adults and elderly individuals, with limited data across a full age spectrum. This gap constrains the understanding of age-related differences in immune responses to SARS-CoV-2. A more comprehensive study including all age groups is required to examine how age affects the immune system’s response to SARS-CoV-2 variants. Furthermore, previous research has often not distinguished whether antibody responses followed breakthrough infections [14,15,16]. Given the widespread background of hybrid immunity following such infections [17], comparing antibody levels across different age groups after breakthrough infections becomes particularly crucial. Additionally, with the ongoing evolution of SARS-CoV-2, it is imperative to investigate both the cross-immunity and broad-spectrum effectiveness of antibody responses to emerging variants across different age groups. These investigations will play a key role in determining both the susceptibility to and severity of future infections.

To combat the COVID-19 pandemic, China implemented a dynamic ‘zero-COVID-19’ strategy, emphasizing rapid virus containment and comprehensive preventive measures. After maintaining this policy for over two years, the Chinese government officially ended it in January 2023. This policy shift was promptly followed by a sudden epidemic, primarily driven by emerging Omicron variants, resulting in widespread vaccine breakthrough infections and a transition to hybrid immunity among numerous individuals [17]. In this study, a cohort comprising all age groups in China was established following the termination of the ‘zero-COVID-19’ policy. Over the year following the policy change, three follow-up visits were conducted to investigate (1) the dynamics of NAbs against emerging SARS-CoV-2 variants (BA.5, XBB.1.5, EG.5, and JN.1) among children, adult, and elderly individuals; (2) the progression of cross-reactive and broad-spectrum NAbs in these age groups; and (3) the factors influencing both aspects.

## 2. Materials and Methods

### 2.1. Participants and Design

A prospective cohort study was initiated in Hubei Province to examine long-term antibody levels and dynamic health status changes among residents of all ages following the termination of China’s zero-COVID-19 policy and the ensuing COVID-19 wave. A total of 1065 participants were recruited, with 859 individuals completing three visits by October 2023. These visits were conducted in mid-March (Visit 1), mid-June (Visit 2), and mid-October (Visit 3) of 2023. During each visit, participants completed questionnaires to gather baseline and follow-up information. Trained investigators conducted interviews using semi-structured questionnaires to collect data on demographic factors, lifestyles, health status, history of SARS-CoV-2 infection and symptoms, COVID-19 vaccination history, and medical history. SARS-CoV-2 infection prior to Visit 1 was defined as a positive result on polymerase chain reaction (PCR) or antigen testing. Vaccination information, including vaccine type and administration date, was obtained from the COVID-19 vaccination database of Hubei Province. These inactivated vaccines including Sinopharm and CoronaVac COVID-19 vaccines were made using conventional whole inactivated SARS-CoV-2 virus technology and adjuvanted with alum. Blood samples were collected at each visit and subsequently separated into serum, plasma and peripheral blood mononuclear cells (PBMCs). Serum and plasma were stored at −80 °C, while PBMCs were preserved in liquid nitrogen.

**Figure 1:**
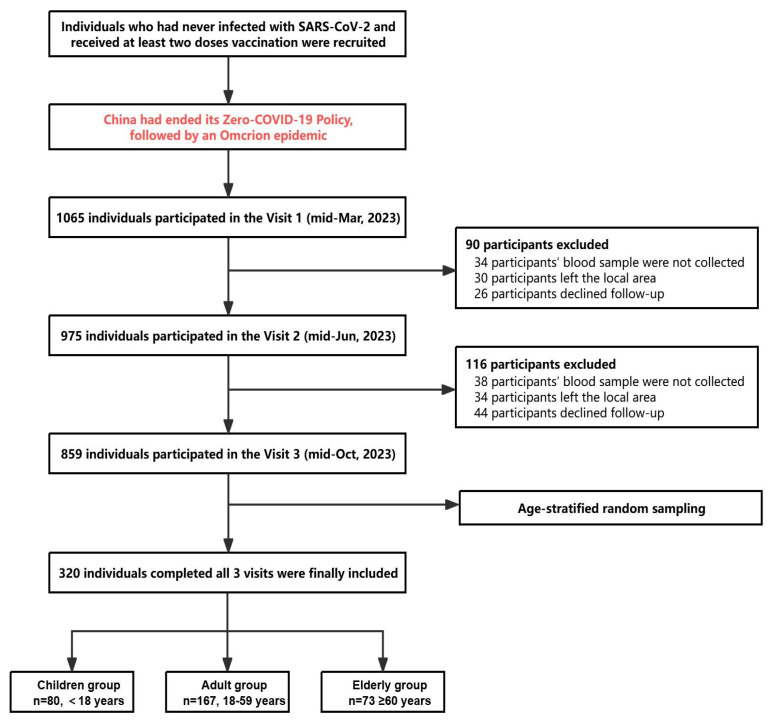
**Overview of participants enrolled in the prospective cohort study.** Participants were enrolled in the prospective cohort study as described in detail in the Materials and Methods section. The primary reasons for participant exclusion included the inability to obtain blood samples, relocation from the study area, and refusal to participate in follow-up assessments. Blood samples were collected from 320 participants and tested for neutralizing antibodies at each visit.

**Figure 2:**
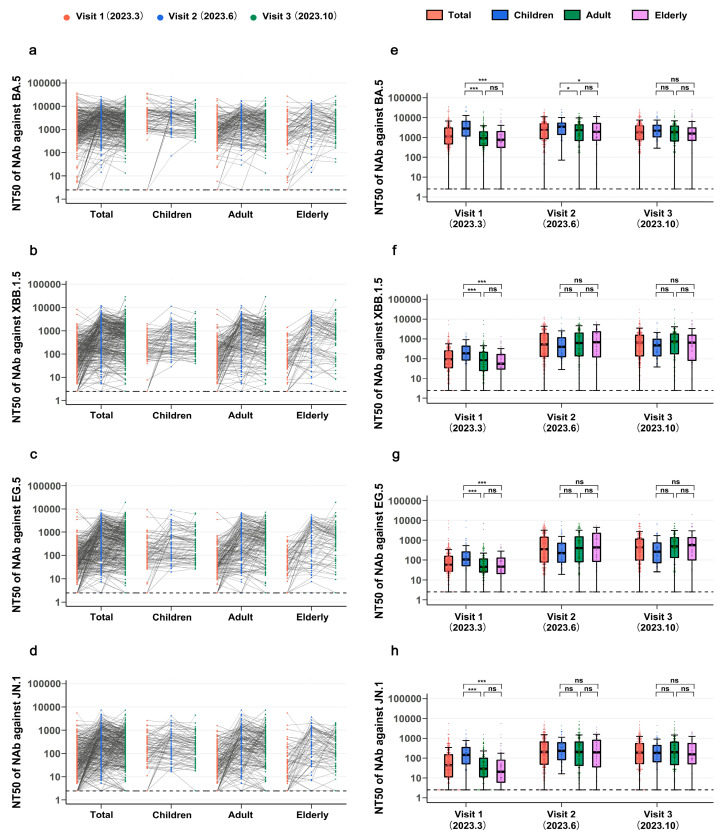
**Longitudinal assessment of neutralizing antibody against SARS-CoV-2 variants across different age groups.** (**a**–**d**) Graph manifesting temporal changes in NAb against BA.5 (**a**), XBB.1.5 (**b**), EG.5 (**c**), and JN.1 (**d**) variants in different age groups from Visit 1 to Visit 3. (**e**–**h**) The boxplots illustrated NAb against BA.5 (**e**), XBB.1.5 (**f**), EG.5 (**g**), and JN.1 (**h**) variants from Visit 1 to Visit 3. Boxplots indicated median values with the 25th and 75th percentiles of the half-maximal neutralizing titer for serum (NT50), and whiskers showed the 1.5 interquartile range. Dashed Line: The lower limit of detection. *p* > 0.05, not significant (ns); * *p* ≤ 0.05; *** *p* ≤ 0.001.

Due to the extended duration and high cost associated with detecting NAbs against multiple emerging variant strains, comprehensive testing of all study subjects was not feasible. Consequently, after excluding participants with self-reported immunocompromised status, incomplete COVID-19 vaccination records, or missing follow-up information, age-stratified random sampling was performed to select 320 participants (Figure 1). Sample-size calculations were performed with PASS (v23_0_6), and the final sample size met the minimum requirements for the relevant statistical tests used. This sample, with ages ranging from 4 to 91 years, comprised 80 individuals under 18 years of age, 167 individuals aged 18–59, and 73 individuals aged 60 or above. These participants were tested for NAbs against the BA.5, XBB.1.5, EG.5, and JN.1 variant strains, with the results incorporated into the study analysis (Figure 2). A comparison of characteristics at Visit 1 between the randomly sampled population and the total population revealed no significant differences (Supplementary Materials Table S1).

### 2.2. Preparation of Pseudotyped Viruses

As previously described [18], we employed a method for the preparation and titration of Vesicular stomatitis virus-based SARS-CoV-2 pseudoviruses. These pseudoviruses harbored spike proteins derived from Omicron variants BA.5 (GeneBank ID: UUB67350.1), XBB.1.5 (GISAID ID: EPI_ISL_16729522), EG.5 (GISAID ID: EPI_ISL_18111706), and JN.1 (GISAID ID: EPI_ISL_18878531), with the c-terminal of the corresponding cDNA encoding these proteins truncated by 18 amino acids (aa) and human codon optimized to enhance their expression efficiency in HEK293T cells upon cloning into the mammalian expression vector pCAGGS. Then, HEK293T cells were transiently transfected with the pCAGGS-SARS-CoV-2-spikeΔ18 plasmid and cultured at 37 °C with 5% CO_2_ for 24 h. After removing the supernatant, cells were exposed to Vesicular stomatitis virus-ΔG-enhanced green fluorescent protein for 2 h, washed three times with PBS, and maintained in 2% fetal bovine serum (Gibco, New York, NY, USA) at 37 °C with 5% CO_2_ for another 24 h. The supernatant containing pseudoviruses was harvested, filtered through a 0.45 µm membrane, dispensed into 1.5 mL tubes, and stored at −80 °C.

### 2.3. Serum NAb Detection Based on Pseudotyped Virus Neutralization Assay

To perform the pseudovirus neutralization experiment, serum samples from participants were diluted in six four-fold serial dilution gradients before being mixed with quantified SARS-CoV-2 pseudoviruses. After incubation at 37 °C for one hour, the mixture was added to a monolayer of Vero-E6 cells in a 96-well microplate. The experiment was performed in duplicate, and the final result was obtained by averaging the two measurements. After a further 24 h incubation at 37 °C, luminescence measurements were acquired using an Operetta high-content imaging system coupled with Harmony imaging analysis software (PerkinElmer, RRID:5CR_018809). The half-maximal neutralizing titer for serum (NT50) was determined using nonlinear probability regression analysis (unweighted least squares regression; with constraints: top = 1, bottom = 0). The limit of detection (LOD) for NT50 was set at a titer of 1:5, and if the calculated NT50 value fell below the initial dilution value (1:5), it was assumed to be half of the limit of quantification (NT50 = 2.5).

### 2.4. Statistical Analysis

Data processing and statistical analyses were performed using R version 4.3.2 and SPSS 26.0. Categorical variables are summarized as counts with percentages, and continuous variables as medians (interquartile range, IQR). Baseline characteristics among subgroups (children, adults, elderly, or antibody trajectory groups) were compared using the chi-square or Fisher’s exact test, depending on data distribution. The Kruskal–Wallis test, followed by Bonferroni correction for multiple comparisons, was applied to evaluate differences in NAb titers across subgroups. Spearman’s rank correlation was used to assess the association between age and log-transformed NAb titers. Logistic regression was employed to examine the effects of age and sex on antibody trajectories, with other clinical characteristics included as covariates. Odds ratios (ORs) with 95% confidence intervals (CIs) were calculated, and *p* values were reported.

Group trajectory models were determined using latent mixture models through the PROC TRAJ program in SAS (version 9.4). This approach identifies clusters of individuals with similar longitudinal antibody patterns by fitting a discrete mixture model [18]. Model fit was assessed using Bayesian information criteria (BIC) and the number of participants in each trajectory, as described previously. A two-sided *p* value less than 0.05 was considered statistically significant for rejecting the null hypothesis.

## 3. Results

### 3.1. Baseline Characteristics of Study Participants

A total of 320 participants completed three visits with pseudovirus neutralization testing. The cohort comprised 80 children, 167 adults, and 73 elderly individuals (Supplementary Materials Table S2). The median age was 13 years (IQR 11–14) for children, 40 years (IQR 33–49) for adults, and 68 years (IQR 65–71) for the elderly. Males accounted for 50.0% (40/80), 51.5% (86/167), and 46.6% (34/73) in the respective groups.

All children received two doses of the inactivated vaccine before Visit 1. Among adults, 97.6% (163/167) had received at least three doses, while 95.9% (70/73) of the elderly were similarly vaccinated. Breakthrough infections occurred in 83.1% of all participants at Visit 1, with the highest proportion in children (85.0%). Most infections were mild, whereas long COVID-19 was more frequently reported in adults (13.2%).

### 3.2. Longitudinal Assessment of Antibody Levels Against SARS-CoV-2 Variants Across Age Groups

Neutralization responses against Omicron variants BA.5, XBB.1.5, EG.5, and JN.1 were analyzed in a cohort of 320 individuals across different age groups at three separate visits. For the BA.5 variant, the median NT50 at Visit 1 for the entire population was 1151.7 (IQR: 479.6–3037.7), with detectable NAbs in 95.9% (307/320) of participants. The median titer peaked at Visit 2 (2413.4, IQR: 874.5–4946.2) and subsequently decreased to 1850.5 (IQR: 785.3–3832.6) by Visit 3, although remaining higher than during the Visit 1 period. Similar trends were observed across the children, adult, and elderly subgroups (Figure 2a). Between March and June 2023 (Visit 1 to Visit 2), the predominant SARS-CoV-2 strain in China transitioned from BA.5 and its variants to XBB.1.5 and its variants, with a further shift to EG.5 from June to October. The fluctuations in neutralizing antibody levels suggest an increased incidence of XBB.1.5 infections during this period.

For XBB.1.5, the NT50 values in the entire population at the three follow-up studies were 93.2 (IQR: 35.1–245.0), 515.0 (IQR: 131.3–1863.2), and 621.9 (IQR: 140.7–1533.5), respectively, indicating a significant increase over time. A substantial 5.5-fold increase was observed from Visit 1 to Visit 2, followed by a modest 1.2-fold increase from Visit 2 to Visit 3. Similar changes were observed across age subgroups, although a slight decrease was noted in Visit 3 compared to Visit 2 in the elderly group (Figure 2b). NAbs against EG.5 exhibited changes similar to those against XBB.1.5, with a 5.9-fold increase from Visit 1 to Visit 2 and only a 1.2-fold increase thereafter (Figure 2c). The median NT50 against EG.5 for the three Visits were 60.6 (IQR: 26.9–152.9), 353.3 (IQR: 78.7–1396.9), and 437.0 (IQR: 99.1–1144.8), respectively. The median NT50 against JN.1 initially increased and then decreased, with values of 43.7 (IQR: 11.5–150.6), 205.2 (IQR: 49.0–647.2), and 187.9 (IQR: 52.7–541.8), respectively (Figure 2d).

For BA.5, XBB.1.5, EG.5, and JN.1 variants, significantly higher NAb titers (NT50 levels) were observed in children compared to adults and elderly subjects at Visit 1 (*p* < 0.001). However, no significant difference was detected between adult and elderly groups, although NT50 levels in the elderly were generally lower. At Visit 2, children exhibited significantly higher NT50 levels only for the BA.5 variant (*p* < 0.05), with no significant differences observed for other variants. By Visit 3, statistical significance in NT50 levels across age subgroups was no longer evident (Figure 2e–h, Supplementary Materials Table S3). Interestingly, regardless of statistical significance, NAb titers against the BA.5 variant consistently remained highest in children across all three follow-up visits, followed by adults, and were lowest in the elderly. Conversely, for XBB.1.5 and EG.5 variants, children demonstrated the lowest NAb titers at Visits 2 and 3, while the elderly group exhibited higher median NAb titers against EG.5 than the adult group at Visit 3. Regarding the JN.1 variant, median NAb titers in each age subgroup at Visits 2 and 3 followed patterns of children > adults > elderly and adults > children > elderly, respectively. These observed fluctuations in NAb levels against Omicron variants across different age groups and follow-up visits indicate substantial variations in cross-reactivity and infection rates within these cohorts.

### 3.3. Correlation Between Age and NAbs Against Different SARS-CoV-2 Variants

**Figure 3:**
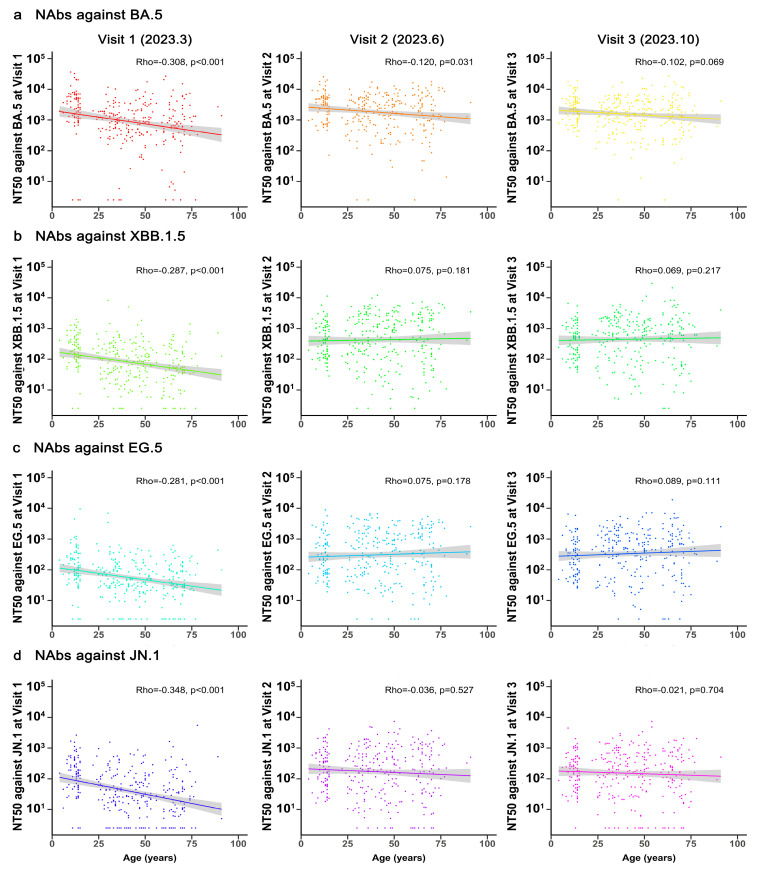
**Correlation between age and neutralizing antibodies against Omicron subvariants across Visit 1 to Visit 3.** A total of 320 participants were included in the analysis. Spearman’s rank correlation coefficients and two-tailed *p*-values are shown in the top right corner.

Further analysis of the correlation between NAb titers and age at Visit 1 was conducted using Spearman’s rank correlation. Results revealed significant negative correlations between NAbs and age for BA.5 (Rho = −0.308, *p* < 0.001), XBB.1.5 (Rho = −0.287, *p* < 0.001), EG.5 (Rho = −0.281, *p* < 0.001), and JN.1 (Rho = −0.348, *p* < 0.001) (Figure 3). Except for BA.5 in Visit 2, no significant correlations were observed between age and NAb levels at Visits 2 and 3, indicating distinct temporal characteristics of NAbs across different age groups.

### 3.4. Longitudinal Dynamics of NAbs Across Age Groups

**Figure 4:**
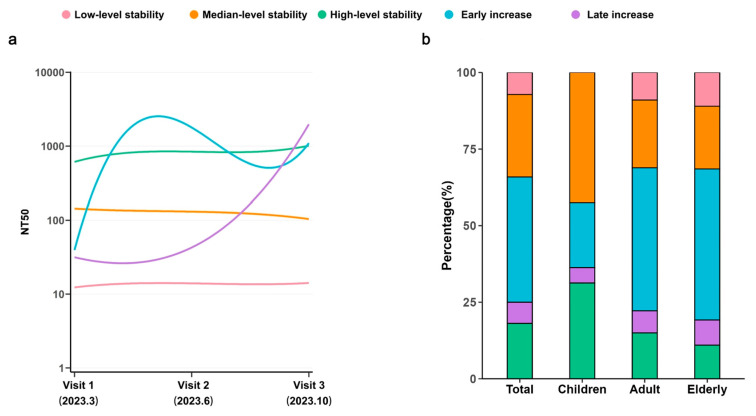
**Trajectory patterns based on neutralizing antibody dynamics.** (**a**) The trajectory patterns of NAbs against XBB.1.5 over time. Based on these trajectory characteristics, patients were categorized into five groups: the low-level stability group (pink), the median-level stability group (orange), the high-level stability group (green), the early increase group (blue) and the late increase group (purple). (**b**) Stacked bar charts illustrating the distribution of the five trajectory groups across different populations.

The longitudinal dynamics of NAbs against XBB.1.5 across different age groups were characterized using group-based trajectory analysis. Five distinct trajectory patterns were identified based on individual changes over three visits (Figure 4a): low-level stability (n = 23, 7.2%), median-level stability (n = 86, 26.9%), high-level stability (n = 58, 18.1%), early increase (n = 131, 40.9%), and late increase (n = 22, 6.9%) (Figure 4b). Among children, the combined proportion of median- and high-level stability trajectories was 73.8%. Conversely, adult and elderly groups exhibited higher proportions of low-level stability groups at 9.0% and 11.0%, respectively. These older age groups also demonstrated higher proportions of early/late increase groups at 53.9% and 57.5%, suggesting elevated infection rates during the period from Visit 1 to Visit 3 for adults and the elderly. Supplementary Materials Table S4 presents the demographic characteristics associated with each trajectory pattern. Age, sex, vaccination status, smoking habits, and infection status were identified as factors contributing to the trajectory pattern composition.

A multivariate logistic regression model revealed that elderly individuals were more likely to exhibit antibody trajectories in the early increase group than in the median/high-level stability group. Male participants, compared with females, had an odds ratio of 0.56 (95% CI: 0.35–0.89) for the early increase group (Supplementary Materials Table S5). Additionally, individuals reporting fatigue had an odds ratio of 1.74 (95% CI: 1.02–2.96) for inclusion in the early increase group and 0.47 (95% CI: 0.28–0.80) for the median/high-level stability group.

### 3.5. Cross-Neutralizing Activity Against SARS-CoV-2 Variants Across Age Groups

**Figure 5:**
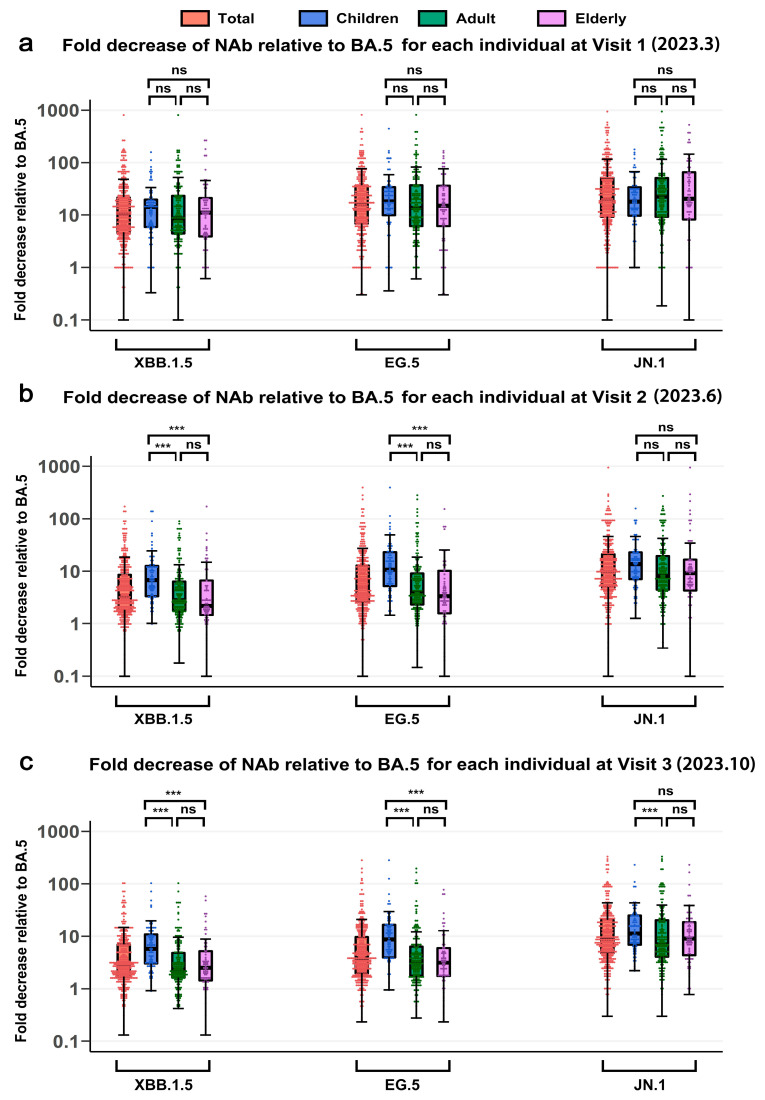
**Comparison of the broad-spectrum efficacy of NAbs against emerging variants across different age groups at Visit 1 to Visit 3.** (**a**–**c**) The calculated fold decrease in NAb titers against the XBB.1.5, EG.5, and JN.1 variants relative to the BA.5 variant for each individual at Visit 1 (**a**), Visit 2 (**b**), and Visit 3 (**c**). The fold decrease was determined as follows: (Fold decrease = NAb titers against BA.5/NAb titers against XBB.1.5, EG.5, or JN.1). Statistical significance is indicated as follows: *p* > 0.05, not significant (ns); *** *p* ≤ 0.001.

Cross-neutralizing activity against SARS-CoV-2 variants was investigated by examining the fold decrease in NT50 of later variants (XBB.1.5, EG.5, and JN.1) compared with the early BA.5 variant across different age groups over three visits. A higher fold decrease indicates reduced cross-neutralization breadth. In Visit 1, there was no significant difference among age groups in the median fold decrease in NT50 for XBB.1.5 (children 14.2, adults 8.6, elderly 11.0), EG.5 (children 18.9, adults 14.1, elderly 15.1) and JN.1 (children 18.4, adults 23.6, elderly 20.5) relative to BA.5 (Figure 5a). At Visit 3, the median fold-decrease in NT50 of XBB.1.5 (children 5.7, adults 2.3, elderly 2.6), EG.5 (children 8.6, adults 3.2, elderly 3.3), and JN.1 (children 11.2, adults 7.0, elderly 9.5) relative to BA.5 was significantly higher in children than in adults or elderly (Figure 5c, *p* < 0.001). These findings indicate a progressively increased risk of infection with newly emerging variants after Visit 3, particularly among children.

## 4. Discussion

This longitudinal cohort study, conducted in China, investigated the dynamics and broad-spectrum effectiveness of NAbs among different age groups following the Omicron epidemic that occurred after the cessation of the zero-COVID policy. Significant age-specific differences in NAb responses were observed. Within the first three months post-epidemic, children exhibited significantly higher NAb levels compared to adults and the elderly. However, this trend reversed after the three-month mark, with a rapid decline in children’s NAb levels. This may be related to the immaturity of the immune system in children. In this age gap, antibody-secreting B cells and plasma cells predominantly drive the immune response to SARS-CoV-2, while conventional memory cells constitute only a small fraction of the B cell response [19]. In contrast, adults and the elderly maintained stable NAb levels beyond three months, possibly due to new infections or reinfections during this period. Consequently, no significant differences in NAb levels were observed between children and other age groups after six to ten months. Notably, the observed NAb dynamics, initially higher in children and subsequently in adults and the elderly, were consistent across various SARS-CoV-2 variants, including BA.5, XBB.1.5, EG.5, and JN.1. Regarding the broad-spectrum effectiveness of NAbs, the effectiveness in children was comparable to that of adults and the elderly within the first three months. However, from six to ten months, children’s effectiveness became significantly lower than that of the other age groups. This suggests that children may perform poorly in responding to newly emerging variants, highlighting the potential necessity of unique immune strategies specifically targeting children.

Previous research has highlighted the importance of NAb levels as indicators for infection prevention and severity [12,20]. Age-related disparities in humoral immunity induced by vaccination or infection have garnered significant attention in recent years. However, considerable heterogeneity exists among studies, with most focusing on vaccine-induced humoral immunity and lacking comprehensive comparisons across all age groups [15,16,21,22,23,24,25,26]. These discrepancies may be attributed to variations in factors such as race, vaccine history, strain type, sample size, and sample collection period across different studies. This study included all age groups and was followed through October 2023, with the advantages of comparable results across age groups and the research population is more representative of the current real world. Our study revealed a negative correlation between age and NAbs against all BA.5, XBB.1.5, EG.5, and JN.1 variants following the BA.5/BF.7 breakthrough infection. This may be attributable to immunosenescence, an age-related decline in immune function in which weakened T-cell and B-cell responses reduce the overall ability to combat infection [27]. This finding provides an explanation for the clinical observation that older adults, whose NAb titres at Visit 1 were the lowest in the cohort, are more susceptible to reinfection compared to younger adults.

A study projected an infection rate of 92.3% (95% CI, 91.4–93.1) by 31 January 2023 [26], while other questionnaire-based studies estimated infection rates of 60–80% in major Chinese cities during the early period following the termination of the “zero-COVID-19” policy [28,29,30]. However, longitudinal studies assessing infection rates over extended periods are lacking. To more accurately estimate infection rates and investigate the longitudinal dynamics of antibodies in the population, five trajectories were identified based on changes in antibody levels over three follow-up periods: low-level stability group (7.2%), the median-level stability group (26.9%), the high-level stability group (18.1%), the early increase group (40.9%), and the late increase group (6.9%). Previous research has demonstrated that breakthrough infections significantly elevate NAb levels. Therefore, the early increase group may be interpreted as those infected from March through June 2023, while the late increase group may represent those infected from June through October 2023.

Our investigation revealed that factors such as age, gender, number of vaccine doses received, breakthrough infection status, and smoking status were associated with the trajectory pattern. Consistent with numerous other studies [15,31], older age and low levels of NAbs were associated with an elevated risk of COVID-19. These findings underscore the importance of increasing vaccination rates among the elderly to reduce COVID-19 incidence. The relationship between gender and COVID-19 remains a subject of debate, with some studies indicating a higher incidence in males [32,33] while others demonstrate the opposite trend [30]. This discrepancy may be attributed to differences in occupational and age structure within the population [30]. In our study, female participants were more likely to be infected with SARS-CoV-2. To be specific, no gender disparity in infection was observed among children (22.5% for females and 20% for males) or elderly individuals (48.7% for females and 50% for males). However, a higher percentage of infection was noted among adult females (59.3%) compared to males (34.9%).

Previous research has demonstrated that hybrid immunity elicits a more robust immune response compared to vaccination or infection alone. Furthermore, individuals who are fully vaccinated and subsequently infected with an earlier Omicron variant have been shown to possess sufficient protection against reinfection with BA.4 or BA.5. However, even in individuals with hybrid immunity, a booster dose or BA.5 infection may not be sufficient to prevent reinfection with XBB [34,35]. Protection against reinfection has been found to diminish over time following the initial infection [36]. Our study reveals that, despite the hybrid immunity of the population after the BA.5/BF.7 breakthrough infection, neutralization activity against XBB.1.5, EG.5, and JN.1 variants was significantly reduced. Consequently, the low neutralizing potency against these later variants may not be sufficient to prevent reinfection. Moreover, in accordance with previous studies [21,22,37], it was observed that children exhibited a narrower breadth of neutralizing antibodies compared to adults, which was also obtained in our study. Despite initially having high levels of NAbs at Visit 1, which were associated with a lower subsequent infection rate, no significant difference in NAb levels was observed between children and the elderly group at Visit 3. In fact, children showed slightly lower NAb levels at this stage, accompanied by a decline in broad-spectrum neutralization capacity. These findings highlight the necessity of enhanced attention to children in the context of SARS-CoV-2 immune monitoring and intervention strategies.

Herd immunity, whether acquired through vaccination or natural infection, has been recognized as a crucial barrier to halting virus transmission [38]. Several WHO—approved vaccines employing various platforms have demonstrated excellent protection against COVID-19 [39]. However, vaccine-induced immunity diminishes rapidly, prompting the U.S. Centers for Disease Control and Prevention (CDC) to recommend a booster dose every 6 months [39]. While a homologous booster dose has been shown to further enhance SARS-CoV-2-specific antibodies [40], significantly improve neutralizing activity against early Omicron variants [14], and effectively prevent COVID-19-related deaths and serious complications [41,42], the Omicron variants have shown a pronounced ability to evade immune barriers enhanced by vaccination and natural infection [3]. Due to immune imprinting, full vaccination with prototype-based vaccines may limit antibody breadth [43]. Fortunately, studies have indicated that Omicron-based vaccines can induce a broad spectrum of NAbs against SARS-CoV-2 [44], underscoring the necessity for novel vaccine development in response to emerging variants.

This study stands out for its prospective design, long-term follow-up after breakthrough infections, large sample size, and systematic serial sampling. It provides an in-depth characterization of the NAb responses against a range of emerging SARS-CoV-2 variants. A notable strength of this study is the comprehensive comparison of NAb responses across all age groups, from children to the elderly, which enhances our understanding of how age influences immune responses to SARS-CoV-2 variants. However, several limitations should be acknowledged. First, B cell responses after breakthrough infection and during hybrid immunity were not evaluated, which may affect the interpretation of the long-term immunity landscape. Second, despite three follow-up visits over ten months after breakthrough infection, the follow-up duration was insufficient to observe a substantial number of reinfections. Further research is required to explore the long-term durability of NAb responses in the context of hybrid immunity. Third, the study population was limited to Hubei Province, which may not fully represent the entire Chinese population. Lastly, the diagnosis of some new Omicron infections during the follow-up was inferred from changes in NAb levels, particularly as the number of SARS-CoV-2 PCR and antigen tests decreased. Specifically, a significant increase in NAb levels against Omicron between Visit 1 and Visit 3 was indicative of a potential infection.

Understanding the long-term dynamics and broad-spectrum effectiveness of NAbs across age groups is critical for guiding future SARS-CoV-2 control strategies. Children show higher NAb levels shortly after the Omicron epidemic, but these levels decline rapidly. Broad-spectrum NAb effectiveness in children remains lower compared to adults and the elderly. This pattern indicates a higher susceptibility to emerging variants in the pediatric population, emphasizing the need for enhanced reinfection surveillance and age-specific protective measures.

## Data Availability

The raw data supporting the conclusions of this article will be made available by the authors on request.

## Supplementary Materials

The following supporting information can be downloaded at: https://www.mdpi.com/article/10.3390/vaccines13101013/s1, Table S1: Baseline characteristics of sample and population; Table S2: Baseline characteristics of different age groups; Table S3: NAbs against SARS-CoV-2 variants across different age groups in three visits; Table S4: Baseline characteristics of participants by different trajectory patterns.; Table S5: The association of predictors with early-increase and median/high-stability trajectories.
