# Supplementary material for: Age-Specific Differences in the Dynamics of Neutralizing Antibody to Emerging SARS-CoV-2 Variants Following Breakthrough Infections: A Longitudinal Cohort Study
Source: Vaccines (Basel). 2025 Sep 28;13(10):1013. doi: 10.3390/vaccines13101013 (PMC12567657; doi:10.3390/vaccines13101013)
Supplement: Supplementary file 1 [file vaccines-13-01013-s001.zip › vaccines-3754608-supplementary.pdf]

Supplementary Material

Age-specific differences in the dynamics of neutralizing antibody to emerging SARS-CoV-2 variants following breakthrough infections

Table S1. Baseline characteristics of sample and population. 1

Table S2. Baseline characteristics of different age groups. 2

Table S3. NAbs against SARS-CoV-2 variants across different age groups in three visits. 3

Table S4. Baseline characteristics of participants by different trajectory patterns. ... 4

Table S5. The association of predictors with early-increase and median/high-stability trajectories. 5

Table S1. Baseline characteristics of sample and population

Characteristic	Sample (n=320)	Population (n=859)
Age, median (IQR), y	40 (20-59)	41 (31-52)
Sex, male (%)	160 (50·0)	465 (54·1)
Residence, n (%)		
Urban	109 (34·1)	386 (44·9)
Rural	211 (65·9)	473 (55·1)
Smoke, n (%)	53 (16·6)	176 (20·5)
BMI, median (IQR)	22·5 (19·6-25·0)	22·8 (20·5-25·2)
Comorbidity, n (%)	41 (12·8)	201 (23·4)
Vaccination status, n (%)		
Primary vaccination	87 (27·2)	119 (13·9)
Booster vaccination	233 (72·8)	740 (86·1)
Breakthrough infections[A]， n (%)	266 (83·1)	753 (87·7)
Asymptomatic	4 (1·3)	15 (1·7)
Mild	240 (75·0)	669 (77·9)
Middle/Severe	22 (6·9)	69 (8·0)
Median (IQR) days from Visit 1 to breakthrough infection	92 (87-96)	92 (86-95)

[A]Breakthrough infection refer to that occurred between post-zero-COVID Omicron epidemic waves and Visit 1.

Table S2. Baseline characteristics of different age groups.

Characteristic	Total	Children (<18 years)	Adult (18-59 years)	Elderly (≥60 years)
n	320	80	167	73
Age, median (IQR), y	40 (20-59)	13 (11-14)	40 (33-49)	68 (65-71)
Sex, male (%)	160 (50·0)	40 (50·0)	86 (51·5)	34 (46·6)
Residence, n (%)				
Urban	109 (34·1)	9 (11·3)	89 (53·3)	11 (15·1)
Rural	211 (65·9)	71 (88·7)	78 (46·7)	62 (84·9)
Current smokers, n (%)	53 (16·6)	0 (0)	39 (23·4)	14 (19·2)
BMI, median (IQR)	22·5 (19·6-25·0)	19·1 (17·3-21·7)	24·0 (21·4-26·0)	22·0 (19·8-24·7)
Comorbidity, n (%)	41 (12·8)	0 (0)	12 (7·2)	29 (39·7)
Vaccination status, n (%)				
Primary vaccination	87 (27·2)	80 (100·0)	4 (2·4)	3 (4·1)
Booster vaccination	233 (72·8)	0 (0)	163 (97·6)	70 (95·9)
Breakthrough infections[A], n (%)	266 (83·1)	68 (85·0)	141 (84·4)	57 (78·1)
Asymptomatic	4 (1·3)	5 (6·3)	1 (0·6)	3 (4·1)
Mild	240 (75·0)	63 (78·8)	130 (77·8)	47 (64·4)
Middle/Severe	22 (6·9)	5 (6·3)	10 (6·0)	7 (9·6)
Long COVID-19	30 (9·4)	5 (6·3)	22 (13·2)	3 (4·1)
Median (IQR) days from Visit 1 to breakthrough infection	92 (87-96)	92 (87-97·3)	92 (87-96)	93 (86-95)

[A]Breakthrough infection refers to infections that occurred between the post-zero-COVID Omicron epidemic waves and Visit 1.

Table S3. NAbs against SARS-CoV-2 variants across different age groups in three visits.

Omicron variants	Total (n=320)	Children (<18, n=80)	Adult (18-59, n=167)	Elderly (≥60, n=73)
Median (IQR)				
Positive detection, n (%)				
BA.5				
Visit 1	1151·7 (479·6-3038·7)	2855·4 (1178·2-6529·1)	910·5 (396·6-1919·4)	773·0 (321·0-1980·0)
	307 (95·9)	75 (93·8)	163 (97·6)	69 (94·5)
Visit 2	2413·4 (874·5-4946·2)	3500·4 (1423·4-5281·3)	2332·6 (714·0-4382·7)	1935·4 (728·8-5168·7)
	317 (99·1)	80 (100·0)	165 (98·8)	72 (98·6)
Visit 3	1850·5 (785·3-3832·6)	2201·6 (1071·5-4134·4)	1826·8 (674·0-3757·0)	1567·4 (720·6-3074·9)
	318 (99·4)	80 (100·0)	166 (99·4)	72 (98·6)
XBB.1.5				
Visit 1	93·2 (35·1-245·0)	185·6 (84·8-425·4)	83·8 (25·1-209·8)	54·8 (29·8-163·2)
	301 (94·1)	75 (93·8)	160 (95·8)	66 (90·4)
Visit 2	515·0 (131·3-1863·2)	391·5 (127·8-1149·9)	609·8 (142·0-1947·2)	666·0 (125·0-2286·3)
	316 (98.8)	80 (100·0)	164 (98·2)	72 (98·6)
Visit 3	621·9 (140·7-1533·5)	465·4 (140·7-966·0)	729·1 (176·5-1754·5)	630·8 (81·2-1534·8)
	317 (99.1)	80 (100·0)	166 (99·4)	71 (97·3)
EG.5				
Visit 1	60·6 (26·9-152·9)	105·7 (52·4-253·1)	45·7 (25·0-113·1)	46·5 (21·3-125·2)
	292 (91.3)	76 (95·0)	155 (92·8)	63 (86·3)
Visit 2	353·3 (78·7-1396·9)	221·6 (77·5-701·0)	401·3 (81·9-1476·2)	435·4 (85·4-2190·8)
	314 (98.1)	80 (100·0)	163 (97·6)	71 (97·3)
Visit 3	437·0 (99·1-1144·8)	257·8 (74·1-728·4)	492·2 (133·2-1335·7)	562·5 (100·6-1355·9)
	314 (98.1)	80 (100·0)	164 (98·2)	70 (95·9)
JN.1				
Visit 1	43·7 (11·5-150·6)	146·3 (51·7-344·0)	28·9 (11·4-99·7)	20·4 (6·0-78·5)
	272 (85.0)	74 (92·5)	141 (84·4)	57 (78·1)
Visit 2	205·2 (49·0-647·2)	231·2 (84·0-592·0)	205·2 (42·9-640·8)	195·4 (36·2-802·6)
	306 (95.6)	80 (100·0)	159 (95·2)	67 (91·8)
Visit 3	187·9 (52·7-541·8)	183·2 (65·2-433·3)	198·9 (47·1-626·2)	156·4 (51·6-542·8)
	301 (94.1)	79 (98·8)	155 (92·8)	67 (91·8)

P values were calculated applying a Kruskal-Wallis test, comparisons were made in the three age groups.

Table S4. Baseline characteristics of participants by different trajectory patterns.

Basic Characteristic	Trajectory					*P* value
	Low-level stability	Median-level stability	Early increase	Late increase	High-level stability	
No. of participants (%)	23 (7·2)	86 (26·9)	131 (40·9)	22 (6·9)	58 (18·1)	
Age						<0·001
<18 years	0 (0)	34 (39·5)	17 (13·0)	4 (18·2)	25 (43·1)	
18-59 years	15 (65·2)	37 (43·0)	78 (59·5)	12 (54·5)	25 (43·1)	
≥60 years	8 (34·8)	15 (17·5)	36 (27·5)	6 (27·3)	8 (13·8)	
Sex						0·003
Male	19 (82·6)	49 (57·0)	55 (42·0)	12 (54·5)	25 (43·1)	
Female	4 (17·4)	37 (43·0)	76 (58·0)	10 (45·5)	33 (56·9)	
Vaccination status						<0·001
Primary vaccination	1 (4·3)	35 (40·7)	20 (15·3)	5 (22·7)	26 (44·8)	
Booster vaccination	22 (95·7)	51 (59·3)	111 (84·7)	17 (77·3)	32 (55·2)	
Residence						0·101
Urban	5 (21·7)	25 (29·1)	51 (38·9)	4 (18·2)	24 (41·4)	
Rural	18 (78·3)	61 (70·9)	80 (61·1)	18 (81·8)	34 (58·6)	
Any comorbidity						0·19
Yes	4 (17·4)	12 (14·0)	21 (16·0)	1 (4·5)	3 (5·2)	
No	19 (82·6)	74 (86·0)	110 (84·0)	21 (95·5)	55 (94·8)	
Smoke						<0·001
Yes	11 (47·8)	13 (15·1)	21 (16·0)	6 (27·3)	2 (3·4)	
No	12 (52·2)	73 (84·9)	110 (84·0)	16 (72·7)	56 (96·6)	
Breakthrough infections[A]						0·003
Yes	14 (60·9)	74 (86·0)	104 (79·4)	19 (86·4)	55 (94·8)	
No	9 (39·1)	12 (14·0)	27 (20·6)	3 (13·6)	3 (5·2)	

[A]Breakthrough infection refer to that occurred between post-zero-COVID Omicron epidemic waves and Visit 1.

The chi-square test or Fisher's exact test was used for categorical variables.

Table S5. The association of predictors with early-increase and median/high-stability trajectories.

	early increase (n=131)		median/high-level stability (n=144)	
	**OR (95% CI)**	**p value**	**OR (95% CI)**	***P* value**
Age group, years				
<18	1 (ref)	1 (ref)	1 (ref)	1 (ref)
18-59	3·35 (1·8-6·23)	<0·001	0·21 (0·12-0·38)	<0·001
≥60	3·62 (1·78-7·37)	<0·001	0·16 (0·08-0·33)	<0·001
Male sex	0·56 (0·35-0·89)	0·015	1·11 (0·70-1·78)	0·66
Smoke	0·95 (0·47-1·94)	0·897	0·53 (0·25-1·13)	0·099
Without comorbidity	0·85 (0·41-1·78)	0·667	0·81 (0·37-1·74)	0·581
Breakthrough infections[A]	0·55 (0·3-1·03)	0·064	2·69 (1·34-5·40)	0·005
Days past infection[B]	1·00 (0·97-1·01)	0·642	1·01 (0·99-1·03)	0·365
Baseline symptoms				
Fever	1·19 (0·64-2·23)	0·579	0·98 (0·53-1·83)	0·959
Fatigue	1·74 (1·02-2·96)	0·041	0·47 (0·28-0·80)	0·005
Cough	0·86 (0·48-1·52)	0·595	1·64 (0·99-2·70)	0·053
Throat pain	1·14 (0·65-1·98)	0·651	0·93 (0·53-1·62)	0·786
Nasal obstruction	0·62 (0·34-1·12)	0·112	1·32 (0·75-2·35)	0·339
Anhelation	1·49 (0·59-3·80)	0·403	1·05 (0·41-2·69)	0·925
Headache	1·65 (0·93-2·92)	0·085	0·61 (0·34-1·08)	0·092
Muscle soreness	1·17 (0·69-2·00)	0·557	0·68 (0·40-1·16)	0·155
Loss of smell and taste	1·03 (0·58-1·81)	0·922	1·03 (0·58-1·82)	0·921
Long COVID-19	0·70 (0·30-1·61)	0·398	1·13 (0·50-2·55)	0·776
$\log_{10} NAbs$ at Visit 1				
BA.5	0·49 (0·35-0·68)	<0·001	5·01 (2·95-8·69)	<0·001
XBB.1.5	0·25 (0·16-0·38)	<0·001	53·74 (20·95-137·82)	<0·001
EG.5	0·30 (0·19-0·46)	<0·001	10·93 (5·82-20·51)	<0·001
JN.1	0·36 (0·25-0·52)	<0·001	4·46 (2·93-6·80)	<0·001

Adjusted for age and gender

[A]Breakthrough infection refer to that occurred between post-zero-COVID Omicron epidemic waves and Visit 1.

[B]Days from Visit 1 to breakthrough infection.
